# Enhancing women’s empowerment to reduce household poverty in rural Western China: social capital as a mediating pathway

**DOI:** 10.3389/fsoc.2025.1638129

**Published:** 2025-10-16

**Authors:** Jing Wang, Rui Gu, Fengying Nie, Thomas Dogot

**Affiliations:** ^1^Agricultural Information Institute, Chinese Academy of Agricultural Sciences, Beijing, China; ^2^Laboratory of Economics and Rural Development, Faculty of Gembloux Agro-Bio Tech, University of Liege, Gembloux, Belgium

**Keywords:** social capital, women’s empowerment, poverty reduction, rural area, Western China

## Abstract

The pathway to household poverty reduction through women’s empowerment is critical for China’s rural revitalization. This study investigated the role of social capital in this process, using data from a household survey conducted in 2021 covering 1,239 dual-adult households in seven historically poverty-stricken counties across four provinces in rural China. We developed social capital indicators using proxy variables from our survey data and predicted the factor scores of the latent variable women’s empowerment. Subsequently, a causal mediation analysis was employed to detect both the direct and indirect effects of women’s empowerment on household poverty. The results have confirmed our hypotheses that women’s empowerment and social capital significantly reduce the probability of household poverty; women with higher empowerment scores are more likely to possess stronger social capital and social capital partially mediates the empowerment–poverty relationship. We concluded that policy-making that aims for sustainable poverty reduction in rural China should prioritize strategies that both strengthen women’s agency and enhance women’s social capital, particularly through structural enhancement measures to expand women’s engagement in cooperatives and associations, strengthen their representation in village governance, foster informal social networks, and invest in women’s education to enhance social capital and sustain poverty reduction in rural areas.

## 1 Introduction

Women’s empowerment is one of the key pillars of sustainable development, encompassing multi-dimensional nature, while the widely accepted content centers on women’s enhanced control and choice-making capabilities. Specifically, empowerment has been defined as both a process and its resultant outcome, through which women gain greater control over material and intellectual resources, and challenge the patriarchal ideology as well as gender-based discrimination against women in all the institutions and structures of society (Batliwala, 1995). Further, empowerment has been conceptualized as a notion of change - “the expansion in people’s ability to make strategic life choices in a context where this ability was previously denied to them” (Kabeer, 1999). Notably, the ability to choose is at the core of the concept of power, and women’s empowerment serves to enhance the ability to exercise choice.

Women’s empowerment plays a significant role in household poverty alleviation. The “feminization of poverty” thesis, which posits that women are disproportionately affected by poverty, has underscored the importance of mainstreaming gender and prioritizing women’s empowerment in poverty alleviation programs (Mackett et al., 2023). Research evidence generally supported that women’s empowerment contributes poverty reduction in rural China. For instance, enhancing women’s decision-making power within families and increasing investment in their education can narrow the income gap between wealthy and poor farmers (Shuai et al., 2019). Portfolio interventions targeting women-including training, credit access and cooperative participation-have been shown to improve the livelihoods of poor households and help them escape poverty (Gu and Nie, 2021). In recent years, technological empowerment via smart phone or internet e-commerce platforms, along with initiatives to promote female entrepreneurship (such as handicraft skill development), has emerged as new channels for rural women to increase their income (Zhu et al., 2022; Jiang et al., 2024).

Social capital, as a key concept in development studies, has clear connotations and multiple authoritative definitions from different academic perspectives. Putnam (1995) conceptualize social capital as networks, norms and social trust that facilitate coordination and cooperation for mutual benefit, which is formed through social connections and civic engagement, and rooted in family and neighborliness. From a multidimensional perspective, social capital can be reflected in three dimensions: the structural one (network ties), the relational one (trust, norms and identification, etc.) and the cognitive one (shared values and narratives)- this framework emphasizes the cultural aspect of social capital (Nahapiet and Ghoshal, 1998). Further, from the structural and relational viewpoint, social capital can be categorized into three types: bonding capital (which fosters social cohesion within peer groups), bridging capital (which establishes structural relationships or networks that cross social groupings), and linking capital (which enables engagement with external agencies that hold power and resources) (Meinzen-Dick et al., 2014). In empirical studies, social capital is often measured using proxy indicators, such as membership in formal or informal organization, ability to get support from those other than family members in case of hardship, gifts given in goods or cash as well as trust in village committees (Abdul-Hakim et al., 2010; Zhang and Zhao, 2025; Guo et al., 2023).

Compared with other well-trodden mechanisms, social capital remains an underexplored yet highly policy-leverageable avenue of research. Case studies illustrated how women create and utilize social capital: for example, becoming a member of a credit cooperatives can help build trust in women, making it easier for them to obtain loans to start a business (Widiyanti et al., 2018). With enhanced agency, women pursue membership and leadership roles in rural cooperatives, cultivate entrepreneurial activities, and expand their participation in community political life-these actions contribute to the accumulation of social capital in the form of trust, networks, mutual support, and cooperation (Saunders and Bromwich, 2012; Chen and Barcus, 2024). From a policy perspective, social capital has been integrated into development policies, including fostering participatory processes (which strengthen cooperation among diverse stakeholders), improving infrastructure and communication technologies (which facilitate broader social connections and knowledge-sharing) (Woolcock and Narayan, 2000). The United Nations Development Program (UNDP) has also incorporated social capital into its framework for guiding rural livelihood improvement, identifying it as social resources such as networks and connections, membership or participation in formal groups, relationships of trust, reciprocity and exchanges that facilitate cooperation and provide the basis for informal safety nets for the poor (United Nations Development Program, 2017). Moreover, research findings confirm that social capital plays a positive role in improving household and residents’ income and increase their welfare, therefore reduces poverty (Syahid and Faathirah, 2021).

Since its first national poverty reduction program—*Priority Poverty Alleviation Program—*was launched in 1994, China has made remarkable strides in poverty alleviation. In 2021, the country officially declared the elimination of absolute poverty, having lifted 98.99 million population out of poverty (State Council Information Office of China, 2021). Over the past three decades, China has also witnessed significant progress in women’s empowerment, particularly in areas such as women’s education, health, income and participation in family decision-making (State Council Information Office of China, 2015). One of the critical goals of China’s Rural Revitalization Strategy is to prevent a large-scale return to poverty. Against this backdrop, a key question arises: how can the potential of women’s empowerment be further unleashed to promote sustainable poverty alleviation? Exploring the potential pathways from women’s empowerment to household poverty reduction is essential to answer this question. To address this gap, this study used the survey data from 19 formerly poverty-stricken villages in western rural China to conduct a mediation analysis focusing on women’s social capital. By doing so, it aims to add new empirical evidence regarding the impact of women’s empowerment on women’s social capital and poverty reduction, while shedding light on the importance of social capital as a mechanism for sustainable poverty reduction.

The rest of the paper is as follows: Section 2 reviews the literature on women’s empowerment, social capital, and poverty reduction and frames the hypotheses for this study accordingly. Section 3 describes the data sources and empirical methodology. Section 4 presents result of the empirical analysis. Section 5 summarizes key findings and discusses the mediation mechanism of social capital. Section 6 is the conclusion touching upon policy implications of this study and topics worthy of deeper exploration.

## 2 Theoretical framework and hypotheses development

### 2.1 Women’s empowerment and women’s social capital

The economic benefits of empowering women have been extensively discussed by researchers since the 1990s. Improving women’s education and health, as well as enhancing their participation in the labor market could increase women’s productivity and income, which in turn raised household income and alleviating household poverty (Morrison et al., 2007). An increase in mothers’ earnings and their decision-making power within the household further facilitates improvements in child welfare, ensuring better education and nutrition outcomes. Consequently, this helped prevent the intergenerational transmission of poverty and mitigates the risk of future household poverty (ibid.). Although definition and measurement variables for women’s empowerment varied, most review studies reported a consistent positive association between women’s empowerment and food and nutrition security (Aziz et al., 2022), which are the fundamental elements in the elimination of poverty. Women’s empowerment, particularly in the sense of bargaining power and decision-making on consumption and human capital investment, has proved to be a strong pathway to poverty reduction through ensuring women’s land rights (Meinzen-Dick et al., 2019). Further, research evidence demonstrated that women empowered by microfinance institutions developed greater entrepreneurship and greater decision-making authority and control over their economic stability, thus reduced their poverty level and also boosted regional economic development (Chen et al., 2025); “left-behind” women in rural China, with enhanced literacy on internet use and e-commerce through government-led training programs, tackled poverty by marketing local agricultural, cultural products (Zhu et al., 2022).

Apart from the direct economic benefits of the empowering women, the social benefits also gained interest of researchers so that the relations between women’s empowerment and social capital were explored. Effective women’s empowerment requires the breaking of gendered social norms, such as the recognition of women’s access to resources, opportunities, and decision making (Galiè et al., 2022), the shifting of perceptions on reproductive work as petty and low-skilled (Singh et al., 2018). Interventions that promoting the family and economic benefits of women’s empowerment could lead to behavior change and alternative social expectations for both men and women thus bringing about some level of norm changes (Hillenbrand and Miruka, 2019). It was argued in a qualitative study that well-designed micro banks that genuinely empowered women—such as those granting women freedom and direct control over the use of loans without requiring spousal approval—could foster women’s social bonding and networks, thus encouraging their collective efforts through its extensive networks, such as village organizations, and community development associations (Khatun and Hasan, 2015). In addition, the implementation of community-based, education-related development programs improved cooperation and trust within project villages and this effect was particularly pronounced among impoverished groups and was directly correlated to the increased agency and empowerment of women (Janssens, 2010). Women’s cooperatives in Indonesia provided women with opportunities for savings and access to credit; the trust built in women’s cooperatives forms a social capital, which helps women obtain loans to start and expand their businesses (Widiyanti et al., 2018). Further, varying relationships were found between women’s empowerment and different types of social capital, for example, bonding social capital was significantly correlated with women’s participation in agricultural decisions; bridging social capital was applied by women to diversify their training and information sources (Po and Hickey, 2020).

Based on the above, we frame the following two hypotheses:

*H1*: Women’s empowerment is positively related with household poverty reduction

*H2*: Women’s empowerment is positively related with women’s social capital

### 2.2 Women’s social capital and household poverty reduction

Social capital could also benefit poverty reduction. A study in rural Malaysia found that an increase of one unit in social capital reduced the probability of household poverty by 18.3% (Abdul-Hakim et al., 2010). Chinese scholars have identified the resource attributes of social networks— business relationships, political connections, and accessible social organizations—which have made significant contributions to poverty reduction, with residents’ trust in institutions playing a moderating role (Zhang et al., 2017). In Bangladesh, households with lower levels of social networks, norms of reciprocity, and civic engagement were more likely to be poor compared to those with higher levels (Islam and Alam, 2018). The impact of social capital on poverty reduction may not always be direct. Compared to physical and human capital, social capital plays a greater role in improving household livelihoods through the mediating effect of intrinsic motivation, including life satisfaction, confidence in improving future living standards, and the ability to face life’s challenges with resilience (Liu et al., 2020). However, the relationship between social capital and poverty reduction were not always consistent: according to the theories of “externalities” of social capital, richer individuals tend to collaborate to those who possess similarly extensive networks, as such ties yield greater access to information, resources, and influence (Collier, 2002). This mechanism of shared cognition and mutual selection inherently disadvantages the poor, who often lack access to high-quality networks.

The impact of social capital on poverty reduction was also explored from the gender perspectives. Women with higher levels of social capital were found less likely to be consumption poor as social capital ensures access to information and other capital resources that enables households to invest more in either farm or off-farm income opportunities (Sharaunga and Mudhara, 2021). Ul-Hameed et al. (2018) found that the positive effect of social capital could be enhanced where vulnerability factors co-existed, i.e., gender discrimination, shortage of financial resources and less focus on development activities by government, etc., indicating that social capital has more significance for women in poor regions to fight against poverty. Women’s agricultural production organization generated closer bonding between family members and friends, helping each other with collective labor and aiding old mothers by donating agricultural products, thus generating more income for the family particularly for daughters and obtaining food security for the poor (Naughton et al., 2017). It was argued that social capital could stimulate rural women entrepreneurs to achieve business success through the enhancement of entrepreneurship and social innovation abilities, enabling them to increase their incomes and improve the welfare of their families, thus fully participating in solving social problems such as poverty and inequality (Osei and Zhuang, 2020). Social capital was found as a mediating factor for the effectiveness of women’s self-help groups. It was found that more mature groups with established trust and economic security enabled women’s active participation in development interventions compared with the poorer and newer groups (Nichols, 2021). Interestingly, the same study also pointed out the “dark side” of social capital: the confinement of women’s traditional roles as caregivers in the domestic sphere and their relatively low educational level leads to women’s exclusion from high-value social networks. This to some extent illustrated the trade-off role of women’s empowerment in mitigating the dark side of social capital.

The above literature suggested that empowered women—who have higher agency, decision-making power, and resource access—are more enabled to leverage social capital effectively for economic gains. Therefore, we frame the following two hypothesis:

*H3*: Increasing women’s social capital has positive effects on household poverty reduction

*H4*: Women’s social capital mediates the relation between women’s empowerment and household poverty reduction

**Figure 1:**
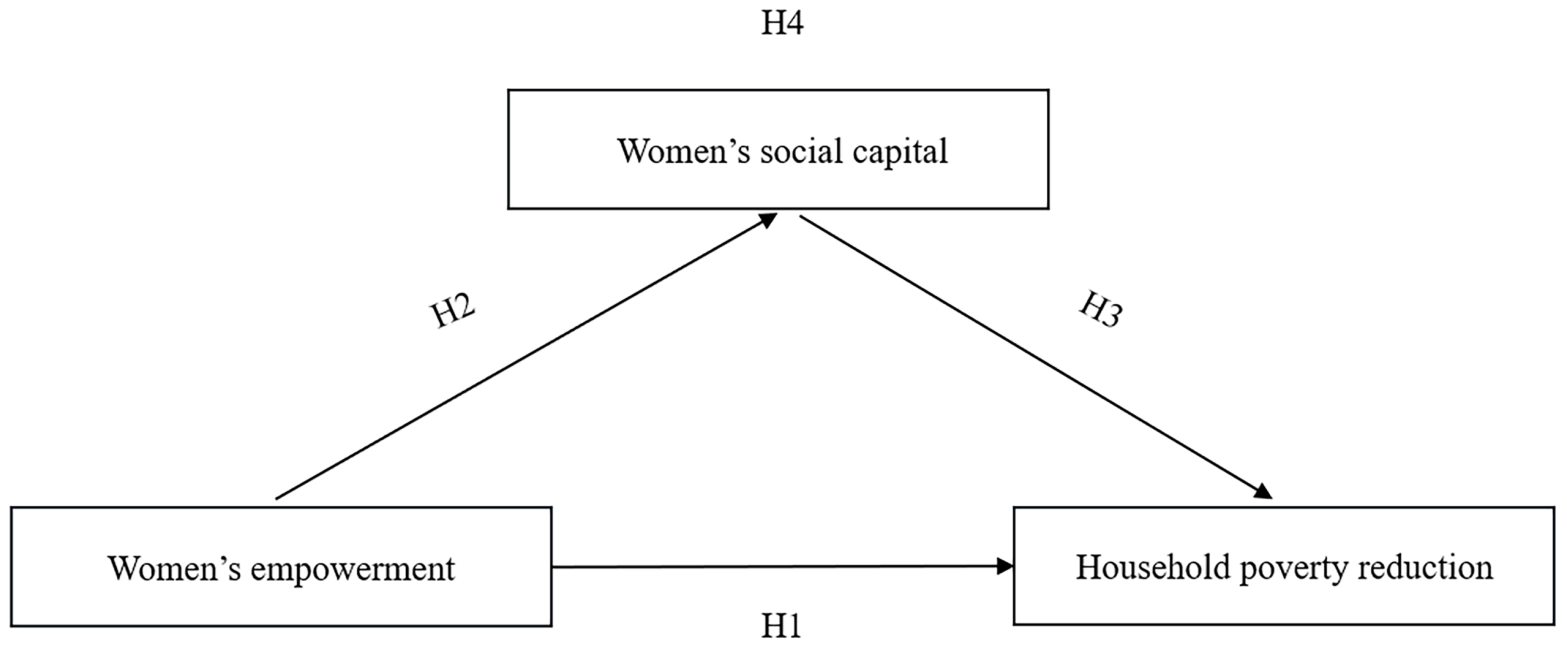
Hypotheses framework.

Figure 1 illustrates the Hypotheses Framework of this paper.

## 3 Material and method

### 3.1 Data sources and sampling

Data used in this study came from a Rural China and Food Security Household Longitudinal Survey in 7 poverty-stricken counties of 4 western provinces of China conducted in 2021. The Survey was started in 2010 and revisited every 3 years. In the 2021 Survey, the research team refined the “women’s empowerment” module with more questions related with social capital, allowing authors to construct the social capital variable for the purpose of this study. In addition, the 2021 dataset provides a timely perspective for analyzing these questions within the broader context of China’s policy transition from elimination of extreme poverty to the prevention of large scale return to poverty.

The 7 surveyed counties were: Zhen’an and Luonan counties in Shaanxi Province, Wuding and Huize counties in Yunnan Province, Panzhou and Zheng’an counties in Guizhou Province and Qingshui county in Gansu Province. The above four provinces held almost one-third of the 592 previously poverty-stricken counties on the 1994 government list for poverty reduction interventions in China’s national program. County selection prioritized the following criteria: alignment with 271 counties identified by Xiao and Nie (2010) as the least food-secure counties, based on a cluster analysis of food security status across 592 poverty-stricken counties; ensuring coverage of diverse agroecological zones in Western China; feasibility of implementation, including the willingness to cooperate of local authorities, although it brought limitations to sampling procedure. 16 or 19 villages in each county were selected through Probability Proportional to Size (PPS) sampling. 12 households were selected randomly in each village.

Data was obtained through interviews with a multi-module questionnaire, including household demographic characteristics, household expenditure, women’s participation in household decision-making as well as women’s group participation and community engagement. We also conducted interviews with village committee leaders with a structured questionnaire on village characteristics, including its transport infrastructure and market access. These data allow us to explore the relationship between women’s empowerment, social capital and household poverty, while controlling for individual, household and village characters.

For this study, we restricted samples to dual-adult households, who had decision-making activities on all selected indicators. This was because household decision-making indicators in the Survey was designed to involve the level of decision input between couples. By restricting the sample to dual-adult households, the study ensures that the analysis captures intra-household bargaining dynamics, which would not be observable in single-adult households. As a result, we got 1,239 samples out of the 1,556 surveyed households surveyed. Caution is warranted when generalizing conclusions to all household types in the study areas.

### 3.2 Measurement of key variables

#### 3.2.1 Women’s empowerment

Kabeer’s notion for women’s empowerment, which encompassed the concepts of resources, agency and outcomes was the most broadly cited one by researchers (Priya et al., 2021). In this study we adopted *agency*-defined as the ability of women to make independent choice and impact on household decision-making as a proxy for women’s empowerment. Drawing on the indicators from Women’s Empowerment Agricultural Index (WEAI) (Alkire et al., 2013), we constructed our measurement of women’s empowerment (WE) using the following indicator variables: (1) decision on farm activities (agricultural production), (2) decision on non-farm activities (livelihood strategies other than agricultural production), (3) decision on assets (to buy or sell furniture, livestock and vehicle), (4) decision on household income (allocation of family budget), (5) decision on social connections (visits to relatives or friends), with best effort to cover the production, control of resources and social connections dimensions of empowerment. Responses were scored according to the following rules: men had the final say alone = 1, men had more final say than women = 2, men had equal final say with women = 3, men had less final say than women = 4, women had the final say alone = 5.

We employed two methods to measure WE. First, we treated women’s empowerment as a latent construct and derived predicted factor scores from a confirmatory factor analysis (CFA) based on the above five observed indicator variables. Second, we calculated a composite empowerment score by summing the values of the five indicators.

#### 3.2.2 Social capital

In this study, we conceptualize women’s social capital as comprising structural and relational dimensions (Osei and Zhuang, 2020), excluding the cognitive dimension due to data limitations. The indicators used to construct the social capital variable reflect bonding, bridging and linking capitals (Panday et al., 2021). Give measurement constraints, we constructed women’s social capital-SOC by summing the binary variables in Table 1. We also constructed a binary SOC indicator-BSOC- (BSOC = 0 if women have none of the following social capital listed in Table 1, BSOC = 1, if women have as least one of the following social capital) for the purpose of testing robustness of the empirical model in this study.

**Table 1:** Types, dimensions and indicators of social capital.

Type	Dimension	Indicators (Yes = 1, No = 0)	Description
Linking	Structural	Loan	Women get loans from the bank
Linking	Structural	Village Congress	Women attend/speak at village congress meetings
Bridging	Structural	Coop_Assoc	Women participate in activities organized by cooperatives, trade association or mutual support groups
Bridging	Relational	Information	Women get information through individual leisure activities and informal social interactions
Bridging	Relational	Volunteer	Women volunteer in community disease prevention
Bonding	Relational	Leader Relative	Women’s relatives are leaders at the village committee

#### 3.2.3 Poverty

Per capita consumption expenditure is widely used as a threshold to define household poverty status. The World Bank introduced two higher poverty line-$3.2 and US$5.5 a day to reflect national poverty lines in lower-middle-income and upper-middle-income economies, respectively, (World Bank, 2018). China is classified as an upper-middle-income country by the World Bank in 2021. Therefore, in this study, we measured household poverty using household consumption expenditure per capita per day and a poverty line of $5.5 in 2011 PPP, or 18.29RMB (2010 constant price, adjusted using 2011 PPP conversion factor and Chinese official consumer price index). If the consumption expenditure is lower than 18.29RMB, poverty = 1, if higher, poverty = 0.

#### 3.2.4 Control variables

As is commonly applied in empirical studies on poverty or farmer’s livelihood (Abdul-Hakim et al., 2010; Tenzin et al., 2015; Shuai et al., 2019), we use women’s age, square of women’s age, women’s education, number of children, household land size, access to bus in the village and access to market in the village as control variables to reflect the individual, household and village level characteristics that may affect household poverty status.

### 3.3 Descriptive statistics

Table 2 is the statistical description of variables for this study.

**Table 2:** Descriptive statistics.

Variables	Mean	Std. Dev.	Min	Max
Outcome variable				
Household poverty (dummy)	0.53	0.50	0	1
Key explanatory variable				
Latent WE	0	0.90	−1.47	2.38
Composite WE	12.62	4.69	5	25
Mediation variable				
SOC	1.96	1.29	0	6
BSOC	0.87	0.34	0	1
Individual-level control variables				
Women age	53.10	11.02	23	84
Square of women age	2,941	1,174	529	7,056
Women education	4.28	3.84	0	16
Household-level control variables				
Number of children	0.86	0.93	0	5
Land size (*mu*)	5.08	4.21	0	17
Village-level control variables				
Access to bus in the village	0.82	0.38	0	1
Access to market in the village	0.21	0.41	0	1
Observations	1,239			

Note: The measurement of latent WE is reported in Table 3.

### 3.4 Model specification

We first estimate a probit model to examine the relationship between women’s empowerment and household poverty:

$Y_{i}^{*}=\alpha_{1}+\beta_{1}{\text{WE}}_{i}+\xi_{1}X_{i}+\varepsilon_{i1},$ $\varepsilon_{i1}∼N(0,1)$


$$Y_{i}=1\{Y_{i}^{*}>0\}$$

Next, we employ a causal mediation analysis (Imai et al., 2010) with social capital as the mediator:


$$M_{i}=\alpha_{2}+\beta_{2}{\text{WE}}_{i}+\xi_{2}X_{i}+\varepsilon_{i2}$$

$Y_{i}^{*}=\alpha_{3}+\beta_{3}{\text{WE}}_{i}+{\mathrm{\gamma M}}_{i}+\xi_{3}X_{i}+\varepsilon_{i3}$ , $\varepsilon_{i3}∼N(0,1)$


$$Y_{i}=1\{Y_{i}^{*}>0\}$$

where $Y_{i}$ =1 indicates that household i is poor; ${\text{WE}}_{i}$ represents women’s empowerment; $X_{i}\;$ includes individual-, household- and village-level controls; $M_{i}\;$ denotes social capital. $\alpha_{1}$ , $\alpha_{2}$ , $\alpha_{3}$ , $\beta_{1}$ , $\beta_{2}$ , $\beta_{3}$ , $\xi_{1}$ , $\xi_{2}$ , $\xi_{3}$ and γ are parameters to be estimated, and $\varepsilon_{i1}$ , $\varepsilon_{i2}$ and $\varepsilon_{i3}$ are error terms.

## 4 Results

### 4.1 Measurement of the latent WE

We performed Confirmatory Factor Analysis (CFA) for the women’s empowerment variable (WE). We got a good fit model by setting the error term of two indicators related, namely decision on farm activities and decision on non-farm activities. The goodness-of-fit indices included the chi-squared test, the root mean square error of approximation (RMSEA), the comparative fit index (CFI), and the standardized root mean square residual (SRMR). According to Nasser-Abu Alhija (2019), we considered a model to be fit if CFI > 0.95, RMSEA<0.08, SRMR<0.06. Although a non-significant *χ*^2^ and a small *χ*^2^/df ratio are also expected for a well-fitting model, it can be exempted if the sample size is big enough. We manually calculated the Composite Reliability (CR) and got 0.778. CR is considered as good it is more than 0.70. Our result suggested satisfactory internal consistency of a construct, meaning the items are closely related and capture the same underlying concept.

Table 3 presents the standardized coefficients (factor loadings) with their standard errors and the value of good fit indices.

**Table 3:** Measurement of women’s empowerment (confirmatory factor analysis).

Decisions	Std. Coef.	Std. Err.	Variances/Covariance	Std. Coef.	Std. Err.
d1	0.659***	0.021	var(e.d1)	0.566	0.027
d2	0.573***	0.023	var(e.d2)	0.672	0.027
d3	0.754***	0.018	var(e.d3)	0.432	0.027
d4	0.597***	0.022	var(e.d4)	0.643	0.027
d5	0.774***	0.017	var(e.d5)	0.401	0.027
			var(WE)	1 (constrained)	
			cov(e.d1,e.d2)	0.256	0.031
Model fit & Reliability	chi2(4) = 25.97, Prob > chi2 = 0.0000				
	RMSEA = 0.067, CFI = 0.989, SRMR = 0.021				
	CR = 0.778				

A new variable (Latent WE) was then created, containing observation-by-observation values of estimated factor scores (meaning predicted values of the latent variable WE). These factor scores were calculated with an analog of regression scoring method following CFA, which combined information from all observed indicators (StataCorp, 2017). The new variable is a continuous one with a mean of approximately zero and a standard deviation of 0.90 (See Table 2).

### 4.2 Effect of women’s empowerment on poverty

To test H1-that women’s empowerment positively contributes to poverty reduction, we got estimates under six model specifications, as presented in Table 4. Columns (1)–(4) adopted a probit approach, with latent WE as the key explanatory variable, and control variables added progressively at women’s individual, household and community levels. Across all these models, the coefficient of latent WE is consistently negative and statistically significant, indicating that higher level of women’s empowerment reduces the probability of a household being poor. The magnitude of the marginal effect has decreased slightly as more covariates are introduced; however, the negative relationship remains robust.

**Table 4:** The effect of women’s empowerment on household poverty.

Y=Poverty	(1)	(2)	(3)	(4)	(5)	(6)
Model	Probit				Probit	IV Probit
WE	Latent WE				Composite WE	Latent WE
	Marginal effect	Marginal effect	Marginal effect	Marginal effect	Marginal effect	Marginal effect
WE	−0.054***	−0.047***	−0.047***	−0.041***	−0.008***	−0.337***
	(0.015)	(0.015)	(0.015)	(0.015)	(0.003)	(0.129)
Women age		0.005***	0.006***	0.006***	0.006***	0.016***
		(0.001)	(0.001)	(0.001)	(0.001)	(0.004)
Women age^2^		0.000**	0.000	0.000	0.000	0.000
		(0.000)	(0.000)	(0.000)	(0.000)	(0.000)
Women		−0.020***	−0.019***	−0.018***	−0.018***	−0.048***
education		(0.004)	(0.004)	(0.004)	(0.004)	(0.010)
Number of			0.072***	0.073***	0.073***	0.194***
children			(0.015)	(0.015)	(0.015)	(0.041)
Land size			−0.001	−0.003	−0.003	−0.014
			(0.003)	(0.003)	(0.003)	(0.009)
Village				0.000	−0.001	−0.033
access to bus				(0.036)	(0.036)	(0.098)
Village				−0.159***	−0.158***	−0.383***
access to market				(0.033)	(0.033)	(0.097)
Observations	1,239	1,239	1,239	1,239	1,239	1,239

Standard errors in parentheses, ***p < 0.01; **p < 0.05; *p < 0.1.

Column (5) replaced latent WE with composite WE in the probit model. The results again reveal a negative and significant relationship between women’s empowerment and household poverty, though with a smaller marginal effect. This finding further confirms the robustness of the relationship.

To address the potential endogeneity concerns, such as omitted variables that may affect both empowerment and poverty, or the reverse causality between the two, we adopted an instrumental variable approach. Here, “decision-making power of a woman’s natal family prior to marriage” was selected as the instrumental variable. Theoretically, this variable is relevant to women’s empowerment: women exposed to female decision-making authority in their natal households are more likely to develop autonomy and bargaining capacity, which in turn shapes empowerment within their marital households. Meanwhile, decision-making power of a woman’s natal family prior to marriage cannot directly affect the current household’s poverty status, satisfying the exogeneity condition.

The first-stage results of the IV-Probit confirm the relevance of the instrument: the coefficient of natal-family decision-making power is 0.194 (*p* < 0.001), which is statistically significant. Additionally, the Wald test of exogeneity (chi2(1) = 3.10, *p* = 0.078) indicates potential endogeneity in the probit model, thereby supporting the preference for the IV specification.

Column (6) presents the IV-probit estimates: the coefficient on women’s empowerment remains significantly negative (*p* < 0.01), confirming that empowerment exerts a causal effect in lowering the likelihood of household poverty. The corresponding marginal effect is substantially larger than the simple probit estimate, underscoring the downward bias when potential endogeneity is ignored.

For control variables, square of women’s age became statistically insignificant once the household- and community-level variables were included in the model. In contrast, women’s education was consistently associated with lower poverty probability. Additionally, a larger number of children in the household increased the likelihood of poverty. At the community level, village access to markets significantly reduced household poverty probability while access to bus services showed no significant association.

### 4.3 The mediation effect of social capital

Two specifications were employed for the causal mediation analysis, corresponding to two measures of social capital: a continuous social capital score (SOC) and a binary social capital variable (BSOC). The results presented in Table 5 reveal two key findings that support the relevant hypotheses. First, women’s empowerment significantly increases women’s social capital (SOC: = 0.211, *p* < 0.01; BSOC: = 0.286, *p* < 0.01), which provides evidence to accept H2. Second, women’s social capital significantly contributes to household poverty reduction (SOC: = − 0.128, *p* < 0.01; BSOC: = − 0.189, *p* < 0.1), and these results further support the acceptance of H3.

**Table 5:** Tests of Hypotheses.

Variables	(1)		(2)	
	M = SOC	Y = Poverty	M = BSOC	Y = Poverty
	Coef.	Coef.	Coef.	Coef.
SOC/BSOC		−0.128*** (0.030)		−0.189* (0.114)
Latent WE	0.211*** (0.039)	−0.088** (0.042)	0.286*** (0.053)	−0.103** (0.042)
Women age	0.012*** (0.003)	−0.016*** (0.004)	0.017*** (0.005)	−0.017*** (0.004)
Women age^2^	−0.001*** (0.000)	0.000 (0.000)	−0.001*** (0.000)	0.000 (0.000)
Women education	0.076*** (0.010)	−0.041*** (0.011)	0.034** (0.014)	−0.049*** (0.010)
Number of children	0.072* (0.038)	0.210*** (0.041)	−0.083 (0.053)	−0.202*** (0.041)
Land size	0.024*** (0.008)	−0.005 (0.009)	0.006 (0.011)	−0.007 (0.009)
Village access to bus	0.009 (0.092)	−0.004 (0.098)	−0.050 (0.126)	0.000 (0.098)
Village access to market	−0.091 (0.086)	−0.450*** (0.093)	−0.172 (0.115)	−0.440*** (0.092)
_cons	1.599*** (0.110)	0.410*** (0.128)	1.154*** (0.151)	−0.368*** (0.154)

1. Standard errors in parentheses, *p < 0.1; **p < 0.05; ***p < 0.01. 2. Women age and age-squared variables were mean-centered to reduce multicollinearity (VIF) in the regression models.

Table 6 summarizes the mediation effects of social capital. Specifically, SOC mediates 23.4% of the total effect of women’s empowerment on poverty, whereas BSOC mediates only 7.1% of this total effect. These results confirm H4: women’s social capital mediates the relationship between women’s empowerment and household poverty reduction.

**Table 6:** The mediation effect of social capital.

Effect	M = SOC	M = BSOC
	Mean	Mean
Average mediation	−0.010	−0.003
Average direct effect	−0.032	−0.038
% of Tot Eff mediated	0.234	0.071

Notably, the substantial gap between the mediation effects of SOC and BSOC underlines a key insight: it is the breadth and density of women’s social networks—not merely their affiliation with a single group—that effectively translates gains in women’s empowerment into sustained household poverty reduction.

## 5 Discussion

Our construct of social capital encompasses both the structural and relational dimensions of social capital, highlighting the importance of two key factors: women’s access to formal social ties, and their ability to leverage diverse relationships to pool resources, coordinate collective action, and exchange information. Notably, the construct further includes three types of social capital—bonding, bridging, and linking—revealing that women draw not only on kinship ties in private spheres but also on broader social interactions in public spaces. Disaggregating the effects of these three distinct strands of social capital remains a task for future research; in the current study, we model their combined impact.

Empirical evidence from our sample demonstrates that empowered women possess more social capital. Specifically, they are more likely to benefit from rural financial networks (e.g., by obtaining bank loans), participate in rural governance (e.g., by joining or speaking at village congress meetings), or engage in activities hosted by cooperatives, associations, or mutual support groups. Such formal ties are embedded with information and opportunities for cooperation, which can be converted into collective power to generate income and new opportunities. For example, the role of social capital in rural cooperatives aligns with findings from previous studies: “farmers’ specialized cooperatives” in rural China provide women with platforms for skills development, trust and reputation building, and accessing market opportunities to boost income (Liang et al., 2014). In Guizhou Province—one of the sample provinces in this study, where every village had rural cooperatives—women who were cooperative members earned more than their migrant husbands through organized tea-picking activities; this economic contribution has also earned them greater respect from family members and the community (Jiang and Huang, 2023). Despite these benefits, fewer than 20% of women in our sample participated in cooperative or association activities, indicating a need for targeted strategies to increase women’s engagement. Similarly, women’s representation in village committees (a form of linking social capital) is critical for expanding their access to public goods and services (Sargeson and Jacka, 2018). However, women’s participation in village governance remains limited in rural China (Yang, 2020). In our sample, while half of the women attended village congress meetings, only one in four expressed their views publicly. This suggests that the potential of this linking social capital— which enables rural residents to connect with and influence higher-level administrative bodies—has yet to be fully unleashed.

Empowered women also leverage informal social interactions, such as leisure activities with friends and neighbors, volunteering in community events, and accessing village leaders through kinship networks. The reciprocity inherent in such informal interactions—occurring in settings like village squares, tea-houses, weddings, and funerals—facilitates information exchange and mutual aid (Li, 2012). Yet our data reveals gaps: only about 20% of women obtained information through informal social interactions, and just 35% volunteered in community activities. This points to a need for strategies to strengthen social cohesion in rural communities.

Additionally, the role of social capital in reducing poverty depends on women’s empowerment as a prerequisite. Although not directly tested in our analysis, broader evidence suggested that empowerment enhances women’s exposure to and inclusion in social networks, alleviating the “exclusion effect” that often limits poor women’s access to social capital. For instance, a case study on the Grameen Bank’s microfinance programs in China argued that repeated efforts to “nudge” women’s empowerment—through reconstructing individual identities and promoting self-governance—foster collective agency, a sense of collaboration, collective action, and mutual support. As a result, the most vulnerable poor women move beyond passivity, beginning to actively engage and interact within their social networks (You and Badertscher, 2024).

## 6 Conclusion

Our findings suggest that empowering women not only directly reduces household poverty but also indirectly contributes to poverty reduction by enhancing women’s social capital. The mediation effect of aggregated social capital from women’s empowerment to poverty reduction accounts for 23.4% of the total effect. This indicates that social capital is a viable mechanism for empowered women to reduce poverty. Based on the above discussion, we suggest policy-making that aims for sustainable poverty reduction in rural China should focus on strategies that both strengthen women’s agency and enhance women’s social capital. Structural enhancement measures are imperative.

First, women’s participation in rural cooperatives and associations should be expanded. This requires not only increasing women’s membership but also a greater number of women in leadership positions and establishing specialized cooperatives in sectors such as tea, handicrafts, and e-commerce to expand women’s market opportunities. Researchers also suggested vibrant cooperatives should deliver benefits beyond income growth, cost reduction, and financial mutual aid, but also take on functions such as community-based elderly care and the education of left-behind children, initiatives that are particularly beneficial to women (He and Yang, 2019).

Second, women’s representation in village governance remains limited, constraining their ability to access public resources. Mandating gender quotas-a mandatory proportion for women’s representation in village committees-could strengthen women’s voices in village decision-making. But such measures shall be consolidated for implementation, particularly in the poor regions, with cautions not to creating “gendered work roles” where women only in charge of family planning, reproductive health or sanitation (Song, 2016).

Third, informal social capital requires deliberate strengthening. Targeted investment is needed in building village community centers and organizing cultural activities to encourage women’s participation and foster stronger reciprocity and cohesion. In our sample areas, women’s age averaged 53 years old, indicating a rather senior population. Evidence showed that elder women participated less in community activities that men as they were often confined to the household domain with housework and caring burdens (Zhong and Cheng, 2022). This highlights the need to design community initiatives that are inclusive of older women, enabling them to engage in public life, exchange information, and access mutual support networks.

Finally, continued investment in women’s education is essential. Both our empirical findings and previous research (Apfeld et al., 2022; Bui, 2025; Spada et al., 2023) consistently demonstrated that education significantly enhances women’s access to social capital and contributes to poverty reduction. Education equips women with skills, confidence, and networks that sustain empowerment over the long term, making it a durable and strategic pathway for poverty reduction in rural areas.

Future research could advance this topic in several ways. First, using more nuanced measurement tools—such as Likert-scale items—to capture the multidimensional nature of social capital and allow for more model specifications. Second, incorporating village-level or community-level measures of social capital could provide a more holistic understanding of how individual empowerment interacts with collective social structures to influence poverty reduction. Third, given the digitalization process in rural China, the roles of social media and rural e-commerce in shaping women’s empowerment, social capital, and household welfare warrant further exploration.

## Data Availability

The datasets presented in this article are not readily available because the data are available from the Agricultural Information Institute of Chinese Academy of Agricultural Sciences upon reasonable request. Requests to access the datasets should be directed to gurui@caas.cn.
